# Association of monocyte and lymphocyte ratio with risk of abdominal aortic calcification

**DOI:** 10.1371/journal.pone.0327888

**Published:** 2025-07-03

**Authors:** Wenting Yan, Tingting Hu

**Affiliations:** Department of Cardiology, The Affiliated Huai’an No.1 People’s Hospital of Nanjing Medical University, Huai’an, China; University of Montenegro-Faculty of Medicine, MONTENEGRO

## Abstract

**Objectives:**

The aim of the study was to evaluate the relationship between monocyte and lymphocyte ratio (MLR) with severe AAC.

**Methods:**

This cross-sectional study enrolled 3041 patients with AAC from the National Health and Nutrition Examination Survey 2013–2014. Abdominal aortic calcification detected with dual-energy X-ray absorptiometry was quantified using the Kauppila score system. We measured white blood cell, neutrophil, lymphocyte counts, monocyte counts, red blood cell, calcium, 25-VitD3, and phosphorus levels in blood samples. Multivariate logistic regression was performed to examine the association between MLR (as a qualitative or quantitative variable) and severe AAC morbidity.

**Results:**

Severe AAC was detected in 273 (9.0%) participants. Multivariate logistic regression analysis indicated that high MLR was an independent predictor of severe AAC (odds ratio [OR] 2.80, 95% confidence interval [CI] 1.04–7.50; *P* = 0.041).

**Conclusions:**

Elevated MLR levels are independently associated with higher odds for severe AAC, which can serve as a new risk factor in clinical practice.

## Introduction

Abdominal aortic calcification (AAC) is highly prevalent in general population, which presents in 22.4% of the male and 16.4% in females, and the prevalence increases to 100% in both males and females over 75 years old [1]. Severe AAC disturbs abdominal aortic blood flow [2] and is associated with a higher risk of major cardiovascular event [3]. In a recent meta-analysis, high-risk patients with advanced abdominal aortic calcification have a higher risk of cardiovascular events, elevated all-cause mortality and in general a poorer prognosis [4].

The pathological studies of vascular calcification have demonstrated that arteriosclerotic calcification, occurring in the neointimal plaque caused by atherosclerosis, has a significant correlation with the progression of AAC [5]. Aortic calcification provides stabilization of abdominal aortic aneurysm growth by reducing aortic wall stress and biomechanical vessel wall stability [6,7].

Inflammation plays a key role in atherosclerosis and vascular calcification. Blood cell ratios, such as neutrophil-to-lymphocyte (NLR) and platelet-to-lymphocyte (PLR), have been proposed as biomarkers of vascular disease severity [8–10]. The monocyte-to-lymphocyte ratio (MLR) reflects the balance between pro-inflammatory monocytes and regulatory lymphocytes and has been associated with adverse outcomes in coronary artery disease and heart failure [11].

However, there is lack of data regarding the association between MLR and severe AAC. Thus, we perform a cross-sectional study to hypothesize that elevated MLR levels, as an elevated inflammatory component, may be associated with unstable AAC and atherothrombotic events.

## Methods

### Study population

**Fig 1:**
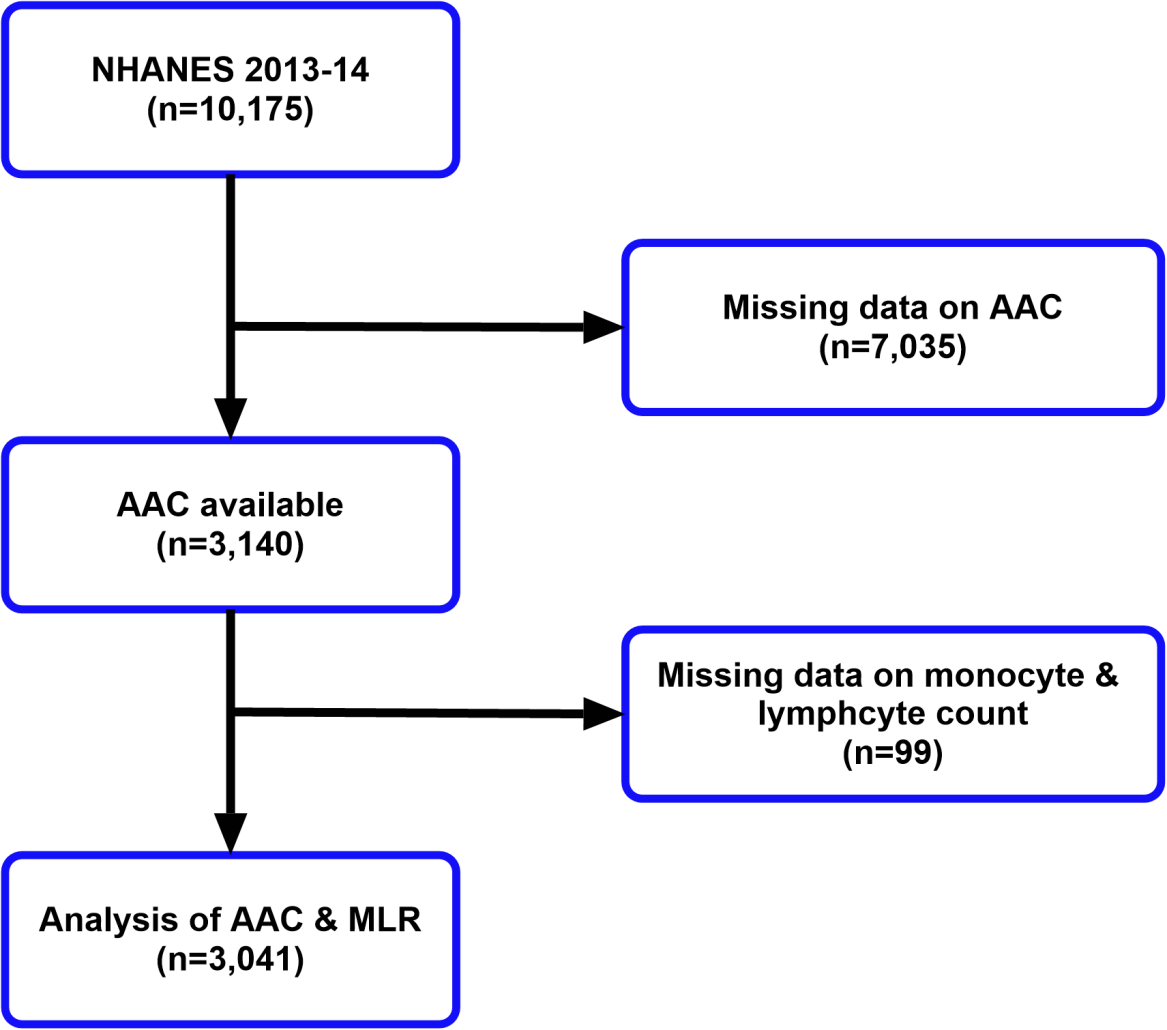
Flow chart of participant selection.

The study included individuals from the National Health and Nutrition Examination Survey (NHANES) 2013−2014. After excluding the missing data on abdominal aortic calcification score, monocyte and lymphocyte counts, 3041 participants were enrolled in our study. Fig 1 depicted the process of participant selection at length. All data used in this manuscript are freely available to the public. Written informed consent was acquired from each participant and the protocol was approved by NCHS Research Ethics Review Board (Protocol #2011-17).

### Survey and measurement

Demographic and health-related data were collected using standardized questionnaires (https://wwwn.cdc.gov/Nchs/Nhanes/2013-2014/DEMO_H.htm). Ethnicity was categorized as Hispanic, White, Black, or Other. Body mass index (BMI) was calculated as weight (kg) divided by height squared (m^2^).Venous blood samples were analyzed for white blood cells (WBC), neutrophils, lymphocytes, monocytes, and red blood cells (RBC) using the Beckman Coulter DXH analyzer. Serum calcium, phosphorus, and 25-hydroxyvitamin D3 were measured using standard biochemical assays. The MLR was calculated as the ratio of monocytes to lymphocytes. Hypertension was defined as self-reported diagnosis, systolic blood pressure ≥140 mmHg, diastolic blood pressure ≥90 mmHg, or use of antihypertensive medication. Diabetes was defined as self-reported diagnosis, fasting glucose >7 mmol/L, HbA1c > 6.5%, or use of hypoglycemic agents.

AAC was assessed via dual-energy X-ray absorptiometry (DXA) of the lumbar spine (L1–L4) and scored using the Kauppila method, with scores >6 defined as severe AAC [12–14].

### Statistical analysis

Data are presented as mean ± SD or number (proportions). According to the median of MLR, two groups were determined. Differences among different MLR groups were explored by T-test or Mann-Whitney U test and chi-square test. Associations between MLR and the risk of severe AAC were estimated by multivariate logistic models with 95% confidence intervals. Multivariate models included some potential covariates including age, gender, race, body mass index, body mass index, systolic blood pressure, smoking, total calcium, VitD3, and phosphorus. The covariables may be involved in calcium and phosphorus metabolism and tissue calcification, or have a significantly distribution across two groups. The associations between levels of MLR and AAC was evaluated on a continuous scale with restricted cubic spline curve based on the logistic models. We also performed subgroup analyses stratified by gender, age, diabetes status, and hypertension. Data were analyzed using IBM SPSS 25.0. *P* value <0.05 was considered as statistically significant.

## Results

The baseline characteristics of patients are presented in Table 1, stratified by presence of severe AAC. Severe AAC was detected in 273 (9.0%) participants totally. Comparing with those without severe AAC, severe AAC patients were older, have higher level of BMI, systolic BP, WBC, neutrophil, monocyte, phosphorus, MLR, and more likely to have lower diastolic BP, lymphocyte, red blood cell, 25-VitD3.

**Table 1:** Baseline characteristics of patients stratified by presence of severe AAC.

Characteristics	Severe AAC		P value
	Yes	No	
n	273	2768	
Female	142 (52.0)	1433 (51.8)	0.989
Age; yrs	71.48 (9.25)	57.35 (11.48)	<0.001
Race			<0.001
Hispanic	34 (12.5)	657 (23.7)	
White	178 (65.2)	1169 (42.2)	
Black	35 (12.8)	548 (19.8)	
Others	26 (9.5)	394 (14.2)	
BMI	27.12 (4.41)	28.59 (5.66)	<0.001
Smoking	60 (31.9)	758 (35.4)	0.373
Waist circumference; cm	98.46 (10.75)	99.41 (13.93)	0.277
Hypertension	204 (74.7)	1235 (44.7)	<0.001
Diabetes	81 (31.5)	422 (15.9)	<0.001
Systolic BP; mmHg	135.59 (20.15)	126.32 (18.47)	<0.001
Diastolic BP; mmHg	62.80 (15.98)	71.73 (12.46)	<0.001
WBC; 1000/ul	7.54 (2.17)	7.11 (2.18)	0.002
Lymphocyte; 1000/ul	1.98 (0.76)	2.10 (0.74)	0.013
Neutrophil; 1000/ul	4.61 (1.74)	4.18 (1.73)	<0.001
Monocyte; 1000/ul	0.66 (0.26)	0.57 (0.19)	<0.001
RBC; 1000/ul	4.40 (0.49)	4.63 (0.47)	<0.001
Calcium; mg/dl	9.48 (0.35)	9.45 (0.37)	0.226
25-VitD3; nmol/l	22.25 (41.22)	31.71 (37.68)	<0.001
Phosphorus; mg/dl	3.87 (0.58)	3.79 (0.57)	0.032
AAC score	11.21 (3.85)	0.69 (1.46)	<0.001
MLR	0.37 (0.18)	0.30 (0.13)	<0.001

Table 2 showed baseline characteristics according to MLR median. High MLR (≥0.28) was detected in 1469 (48.3%). In comparison with those with low MLR level, participants with high MLR level tended to be older, female, have higher level of waist circumference, systolic BP, WBC, neutrophil and phosphorus. There was no significant difference in smoker, diabetes mellitus, DBP and calcium (*P *> 0.05).

**Table 2:** The baseline characteristics of patients according to monocyte-to-lymphocyte ratio.

Variables	Low MLR; < 0.28	High MLR; ≥ 0.28	P value
n	1572	1469	
Female	954 (60.7)	621 (42.3)	<0.001
Age; yrs	56.34 (11.09)	61.06 (12.44)	<0.001
Race			<0.001
Hispanic	423 (26.9)	268 (18.2)	
White	547 (34.8)	800 (54.5)	
Black	325 (20.7)	258 (17.6)	
Others	277 (17.6)	143 (9.7)	
BMI	28.67 (5.62)	28.23 (5.51)	0.030
Smoking	431 (35.0)	387 (35.3)	0.939
Waist circumference; cm	98.71 (13.59)	99.98 (13.73)	0.011
Hypertension	705 (44.9)	734 (50.0)	0.005
Diabetes	269 (17.8)	234 (16.7)	0.455
Systolic BP; mmHg	125.60 (18.26)	128.83 (19.27)	<0.001
Diastolic BP; mmHg	71.35 (11.90)	70.45 (14.22)	0.068
WBC; 1000/ul	7.04 (1.96)	7.27 (2.38)	0.003
Lymphocyte; 1000/ul	2.40 (0.76)	1.76 (0.56)	<0.001
Neutrophil; 1000/ul	3.89 (1.41)	4.58 (1.97)	<0.001
Monocyte; 1000/ul	0.50 (0.15)	0.67 (0.21)	<0.001
RBC; 1000/ul	4.61 (0.46)	4.60 (0.50)	0.372
Calcium; mg/dl	9.46 (0.36)	9.45 (0.38)	0.478
25-VitD3; nmol/l	32.86 (38.01)	28.70 (38.09)	0.004
Phosphorus; mg/dl	3.82 (0.55)	3.77 (0.60)	0.011
AAC score	1.21 (2.84)	2.09 (4.06)	<0.001
Severe AAC	89 (5.7)	184 (12.5)	<0.001

**Fig 2:**
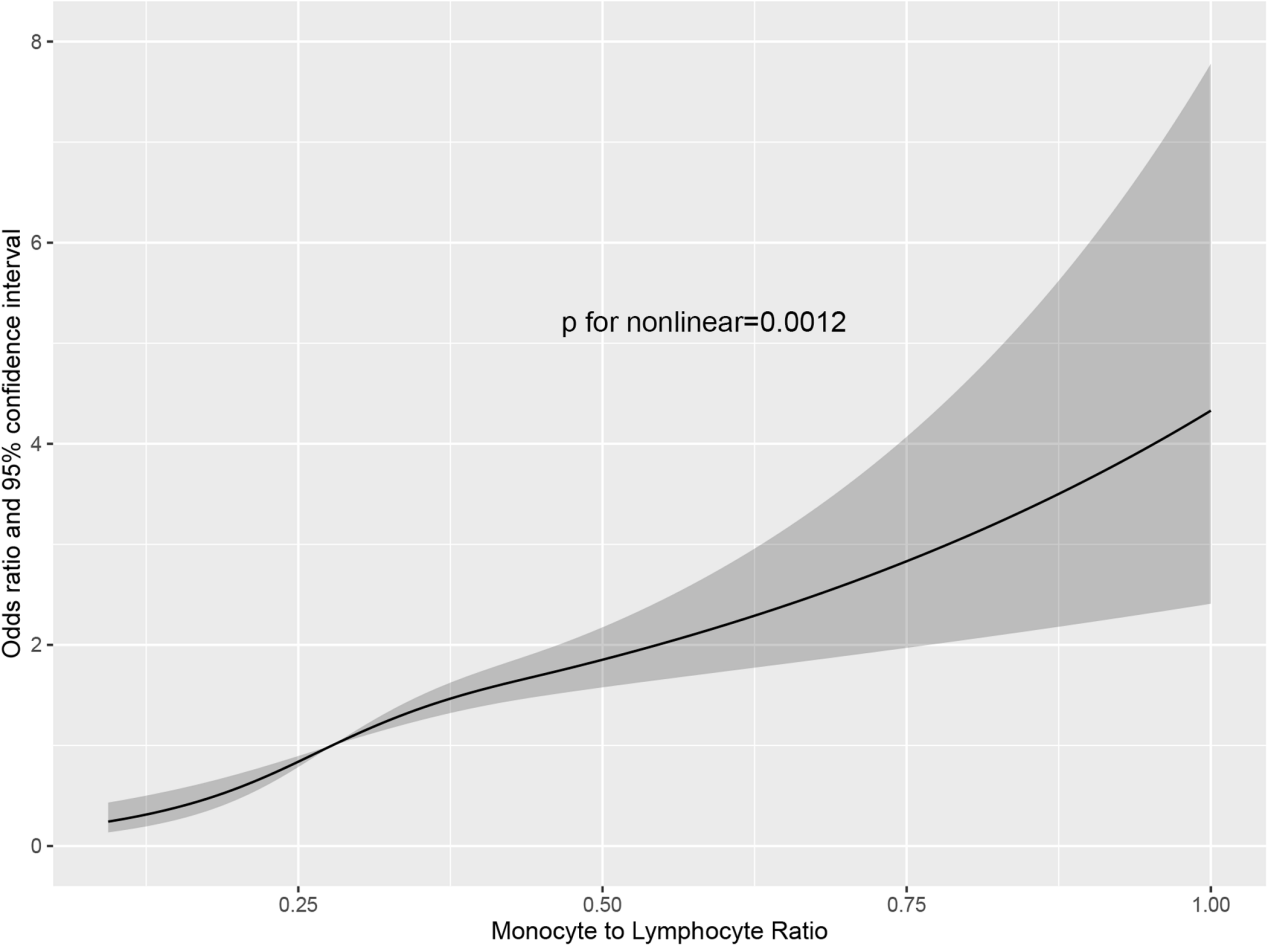
A cubic regression between MLR and severe AAC. Black line is multivariable adjusted odds ratios, with shadow area showing 95% confidence intervals derived from restricted cubic spline regressions.

Logistic regression models were performed to examine the association between MLR level and severe AAC (Table 3). In model 1, after adjusting for age and sex, MLR was found to be positively associated with higher odds of severe AAC (OR:2.96, 95% confidence intervals [CI]: 1.33–6.59; *P* = 0.008). This association was not altered after further adjustment for several other covariates in Model 2 and Model 3, including race, body mass index, body mass index, systolic blood pressure, smoking, total calcium, VitD3, and phosphorus. The multivariable-adjusted odds ratios and 95% CIs of the Model 2 and 3 were 2.94 (1.10–7.91) and 2.80 (1.04–7.50) respectively, *P* < 0.05. What’s more, dose-response analysis revealed a significant nonlinear relation between MLR level and the odds of severe AAC (*P* = 0.0012; Fig 2).

**Table 3:** Multivariable analysis of MLR associated with severe AAC. MLR, monocyte and lymphocyte ratio.

	Model 1	Model 2	Model 3
OR (95% CI)	2.96 (1.33–6.59)	2.94 (1.10–7.91)	2.80 (1.04–7.50)
P value	0.008	0.032	0.041

Model 1 adjusted for age and gender. Model 2 further adjusted for race, body mass index, waist circumference, systolic blood pressure, and smoking. Model 3 further adjusted for total calcium, VitD3, and phosphorus.

**Fig 3:**
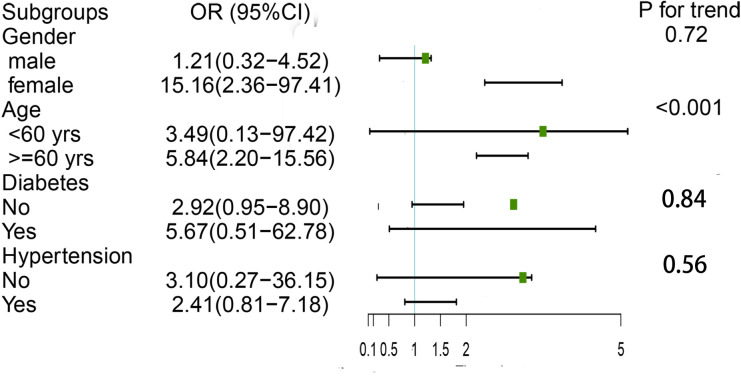
Subgroup analyses of association of MLR with AAC. The boxes represented average OR and whiskers represented 95% confidence intervals of each OR. Age subgroup has interactive effects.

Besides, to examine the consistence of association between MLR and the presence of AAC among subgroups, we performed subgroup analyses. The results were shown in Fig 3. Significant interactions were only found across age.

## Discussion

This study demonstrated a significant and independent association between elevated MLR and the risk of severe AAC. This relationship remained robust after adjusting for traditional cardiovascular risk factors and mineral metabolism markers. Our findings support the role of systemic inflammation in vascular calcification and identify MLR as a simple, accessible biomarker for AAC risk.

Vascular calcification results from the transformation of vascular smooth muscle cells into osteoblast-like cells, compromising vessel elasticity and increasing cardiovascular risk [15,16]. Although some calcification may stabilize aneurysm growth, severe AAC is associated with higher cardiovascular and fracture risk [17,18].

Prior research has linked inflammatory markers like CRP, NLR, and PLR to vascular disease [19,20]. Our study extends these findings by highlighting MLR as an independent and potentially superior marker. Elevated MLR reflects heightened monocyte-driven inflammation and reduced lymphocyte-mediated regulation, both of which contribute to atherosclerosis and calcification [21]. Compared to other markers, MLR offers practical advantages—it is inexpensive, requires no specialized assays, and can be derived from routine blood tests. Its clinical applicability could be particularly valuable for early screening in high-risk populations.

Some limits exist in our study. The cross-sectional design limits causal inference, and our sample is not fully representative of a multi-ethnic or global population, which may impact generalizability. Additionally, incorporating other inflammatory markers, such as CRP, could provide a more comprehensive understanding of the inflammatory profile associated with AAC. Research could also explore how changes in MLR over time correlate with AAC progression, potentially offering a dynamic indicator of vascular health.

## Conclusion

In conclusion, our study underscores MLR as a promising, easily obtainable marker for assessing AAC risk, with potential applications in screening and early intervention for high-risk individuals. Integrating MLR into clinical assessments could enhance AAC management, particularly in older adults and patients with metabolic comorbidities. These findings contribute to a growing recognition of the role of inflammation in vascular calcification and highlight the need for accessible, cost-effective tools in cardiovascular risk stratification.
